# Supplementary figures and images for: Contrasting a reference cranberry genome to a crop wild relative provides insights into adaptation, domestication, and breeding
Source: PLoS One. 2022 Mar 7;17(3):e0264966. doi: 10.1371/journal.pone.0264966 (PMC8901128; doi:10.1371/journal.pone.0264966)

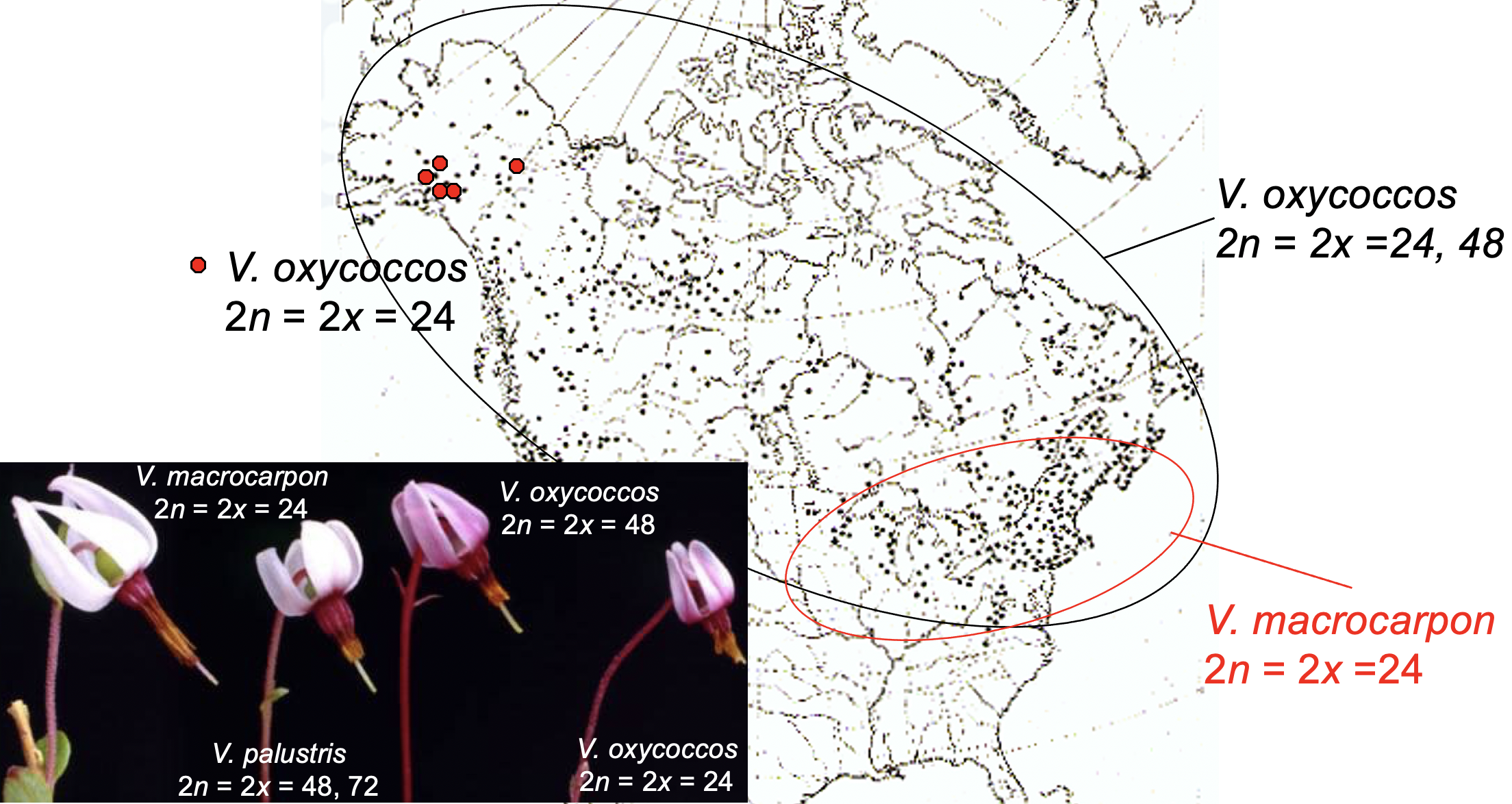

Supplement: S1 Fig — Diploid V. macrocarpon is found in the Northeastern parts of the United States (US), while the diploid V. oxycoccos is found in the Northwestern US and Canada. (TIF) [file pone.0264966.s001.tif]

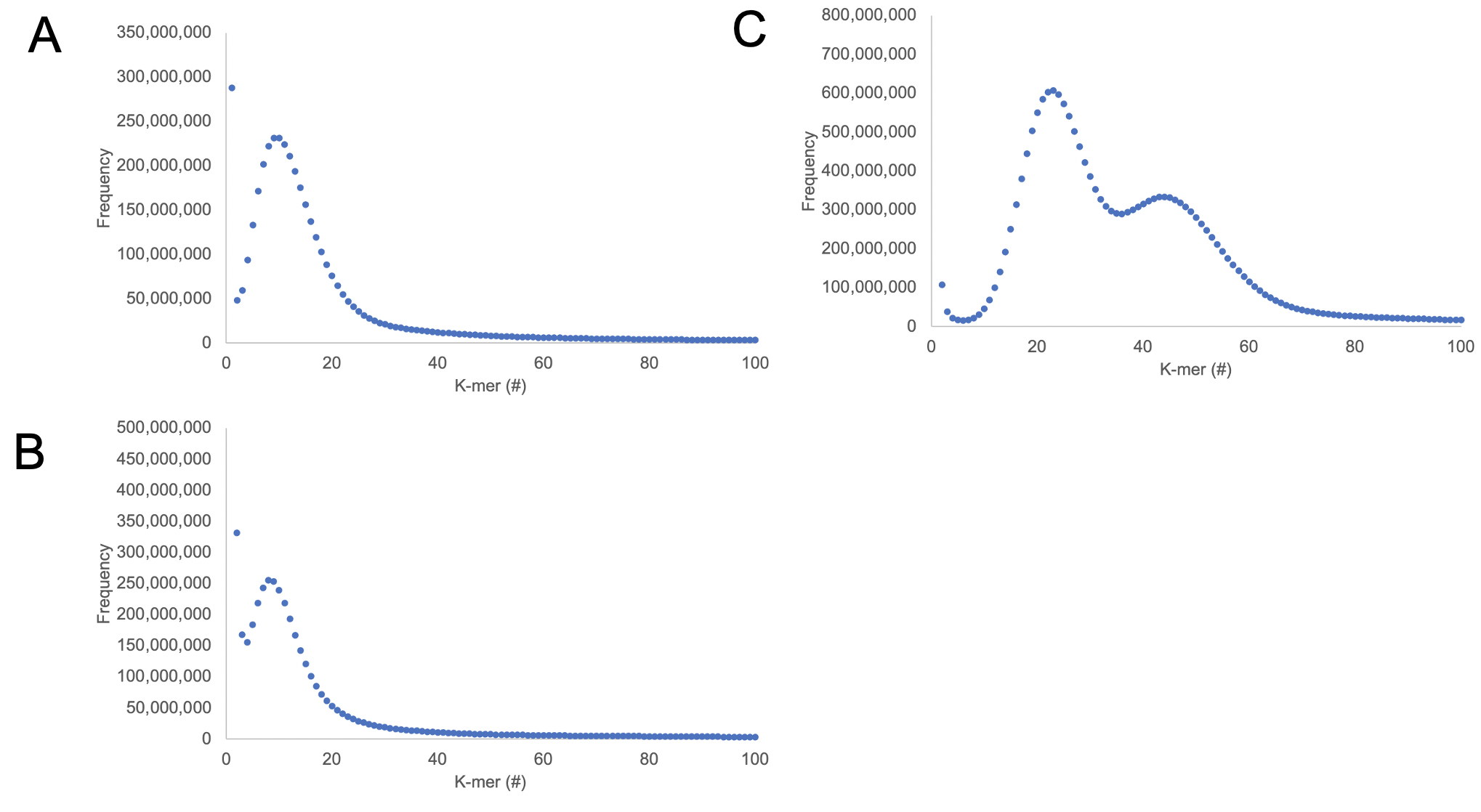

Supplement: S2 Fig — Genome sizes were estimated by K-mer (k = 19) frequency using Illumina paired short reads (2x150 bp) for A) V. macrocarpon (Vmac), B) V. oxycoccos (Voxy), and C) the F1 hybrid. K-mers were counted with Jellyfish and histogram was plotted to find the peak. (TIF) [file pone.0264966.s002.tif]

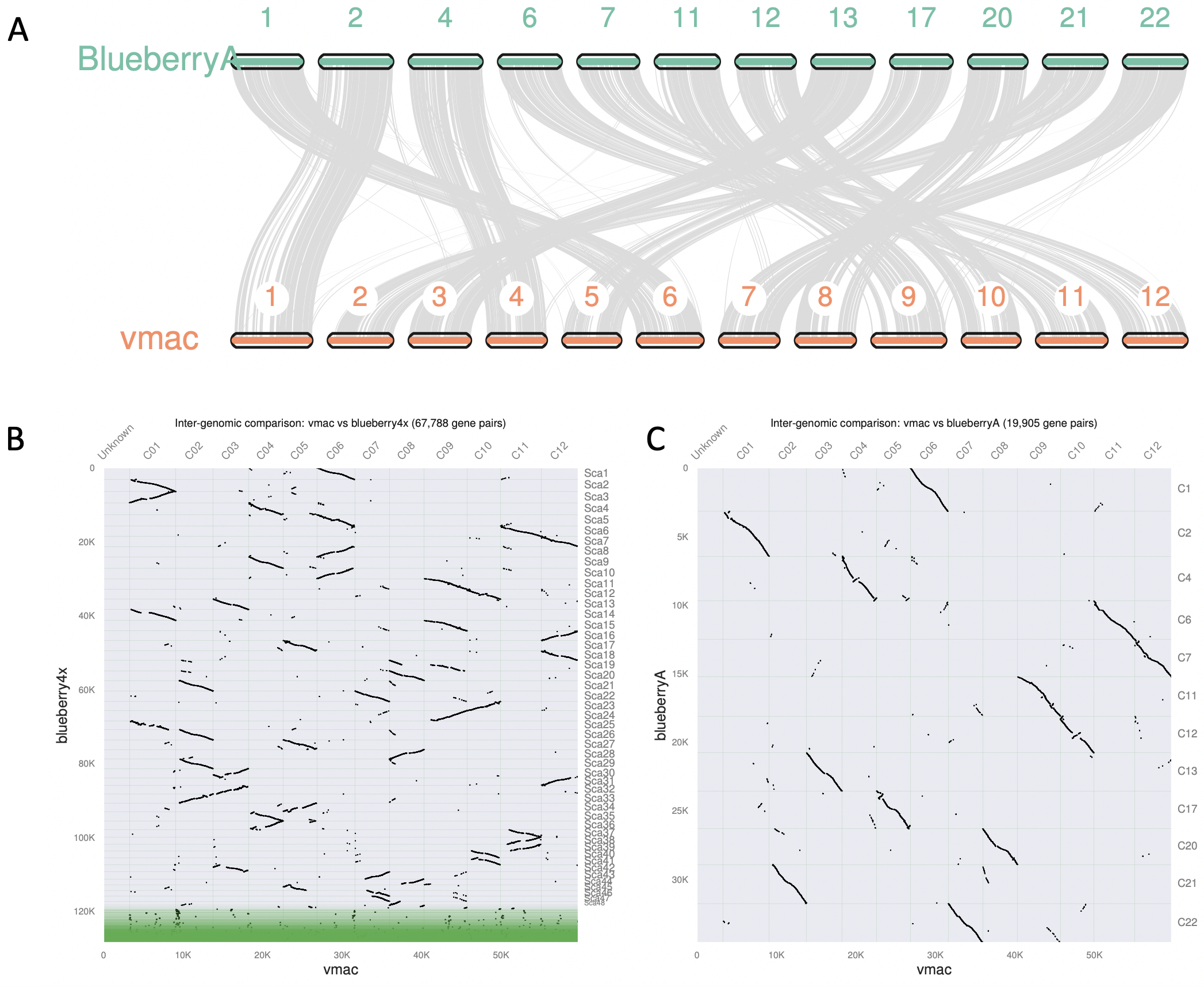

Supplement: S3 Fig — A) Blueberry haplotype A (BlueberryA) was aligned to the Vmac assembly and are presented in the order of their assigned chromosome numbers. B) Dot plot based on protein alignments between the haplotype-resolved tetraploid blueberry (blueberry4x) and Vmac. C) Dot plot based on protein alignments between the blueberry haplotype A (blueberryA) and Vmac. (TIF) [file pone.0264966.s003.tif]

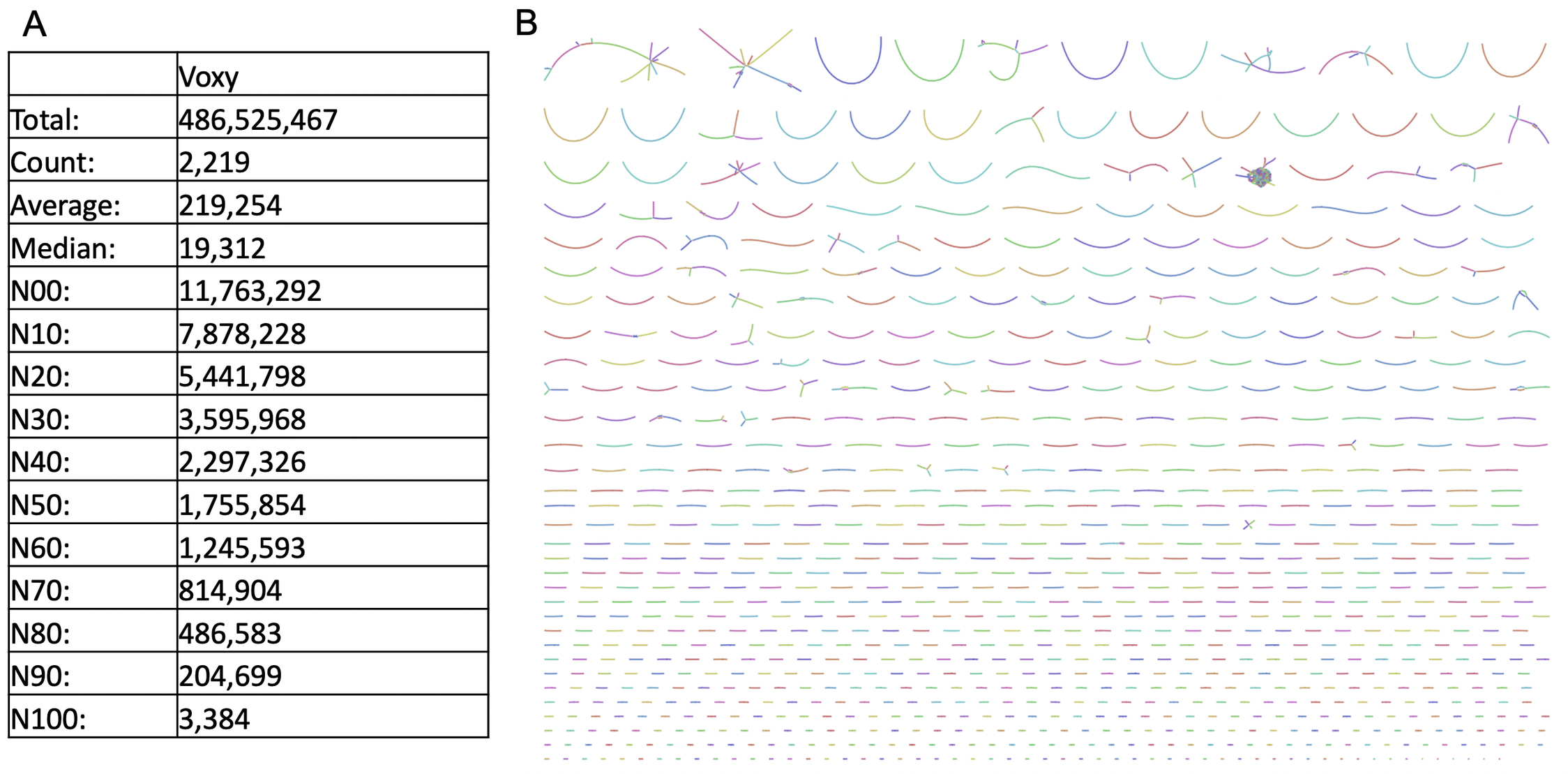

Supplement: S4 Fig — A) Summary of the Voxy contig assembly statistics. The Voxy assembly was 486 Mb, had a N50 length of 1.8 Mb, with the longest contig being 11.8 Mb. B) The assembly graph of Voxy reveals low heterozygosity due to the lack of extensive branching. (TIF) [file pone.0264966.s004.tif]

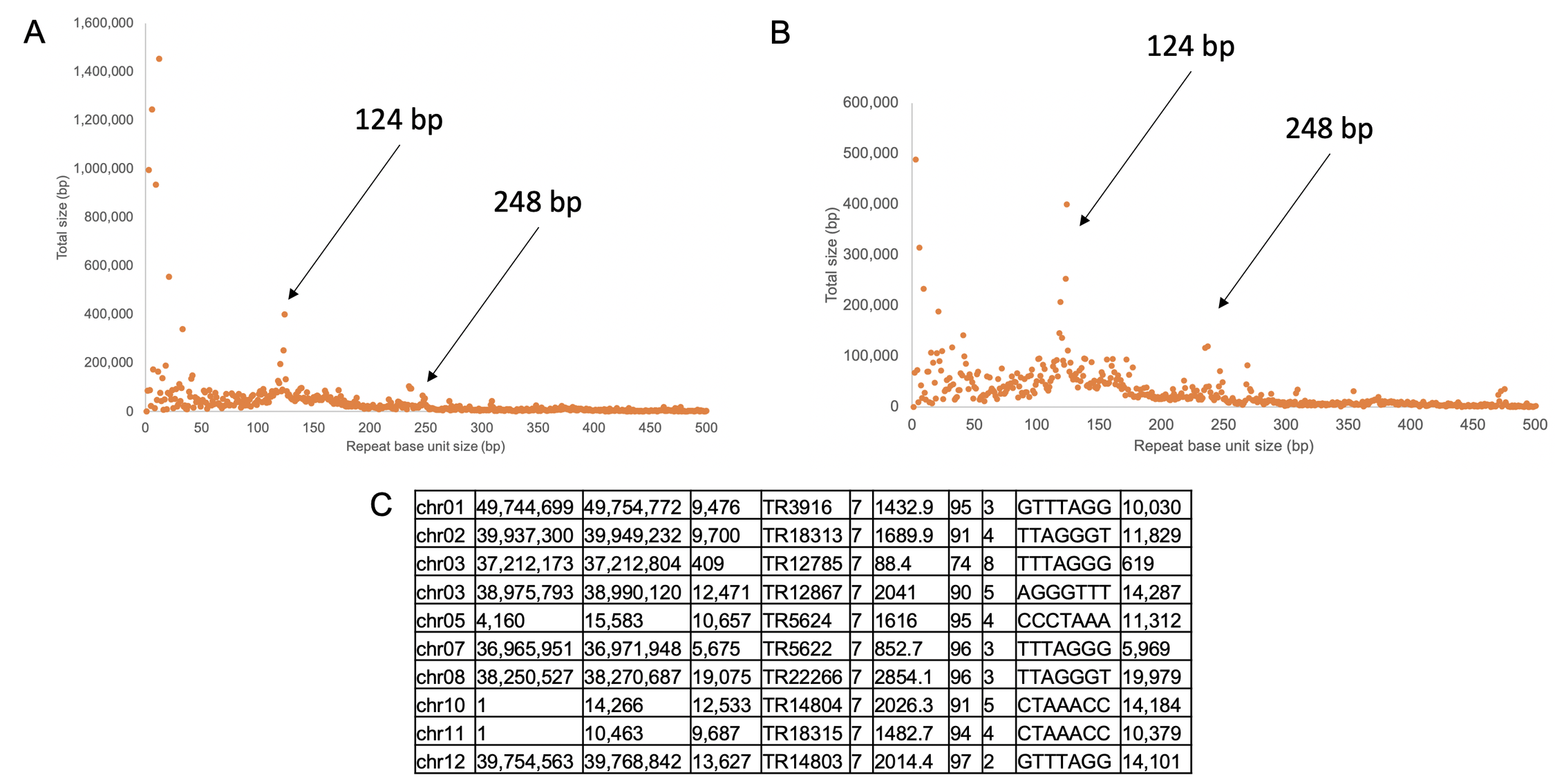

Supplement: S5 Fig — A) Tandem repeats were identified using Tandem Repeat Finder (TRF) and plotted by repeat unit size, which revealed a 124 bp centromere base unit with a 248 bp higher repeat (HOR) consistent with a centromere array. B) A similar centromere array with a base unit of 124 bp and HOR of 248 bp was identified in Voxy. C) Telomere arrays with the 7 bp base unit (AAACCCT) were identified in the Vmac assembly, which revealed an average telomere length of 12 kb. (TIF) [file pone.0264966.s005.tif]

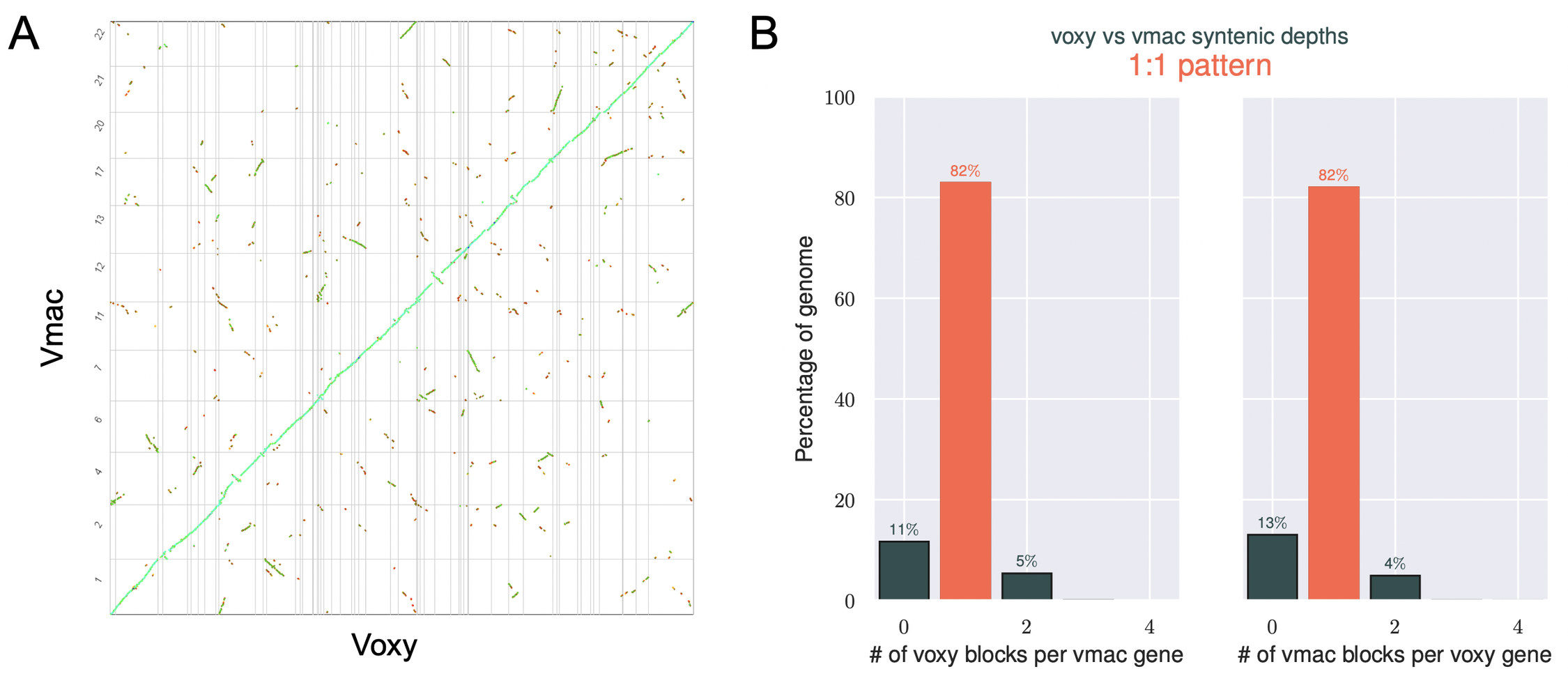

Supplement: S6 Fig — A) The Voxy scaffolds were aligned (protein) to the Vmac chromosomes revealing the two genomes are highly collinear with remnants of a recent whole genome duplication (WGD). Vertical and horizontal grey lines represent breaks in Chromosomes (Vmac) and scaffolds (Voxy) B) Syntenic depths between Vmac and Voxy suggest a 1:1 pattern, although there are remnants of a past WGD at 4–5%. (TIF) [file pone.0264966.s006.tif]

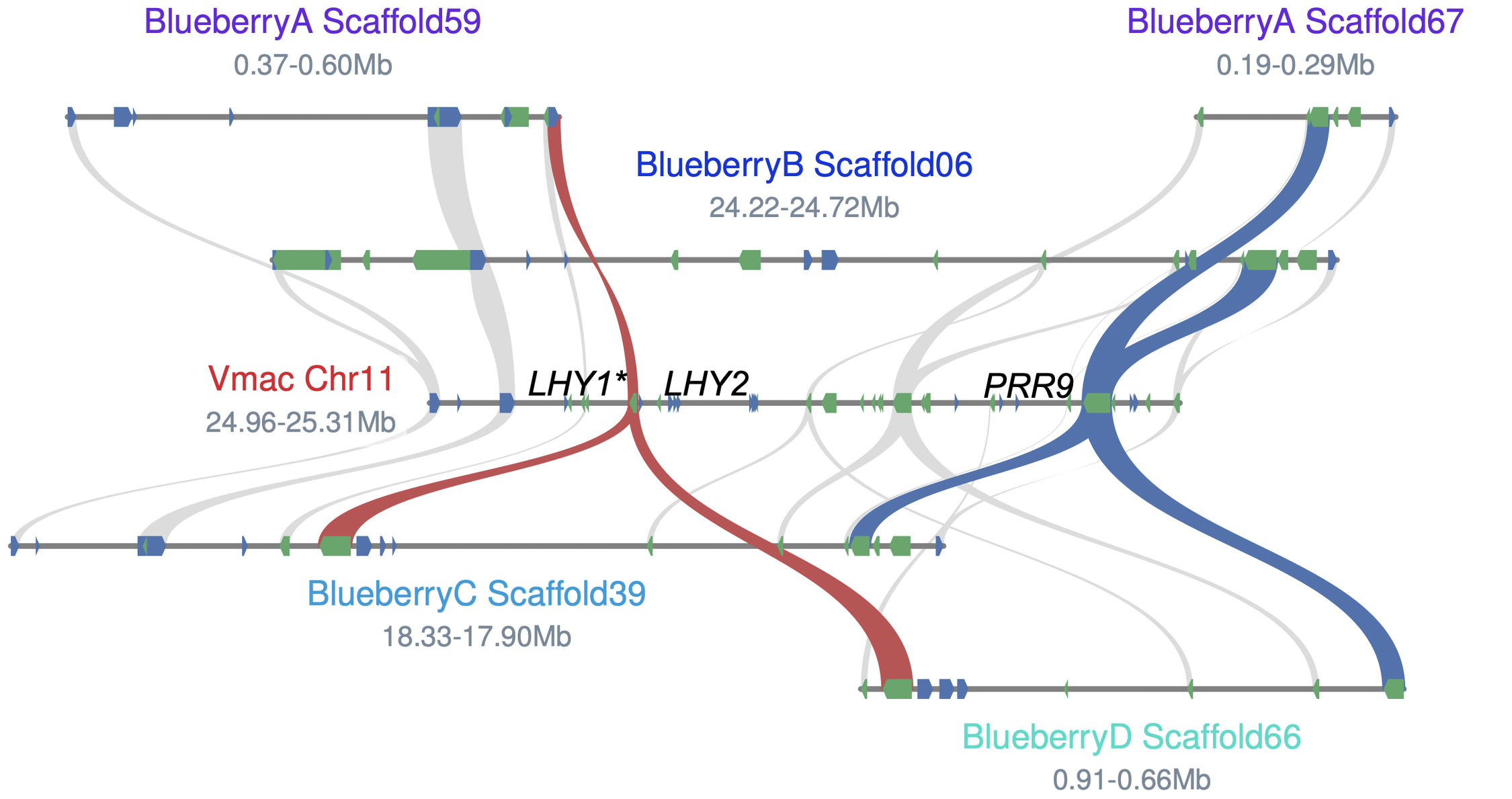

Supplement: S7 Fig — The haplotype-resolved blueberry genome was mapped to the Vmac genome to identify syntenic blocks (grey lines). Blueberry has the core circadian clock linkage of LHY (red lines)-PRR9 (blue lines) on three of its haplotypes, but it has been lost on haplotype B on Scaffold6. The LHY tandem duplication is specific to the Vmac lineage since it is not found in Voxy (Fig 2) nor blueberry. (TIF) [file pone.0264966.s007.tif]

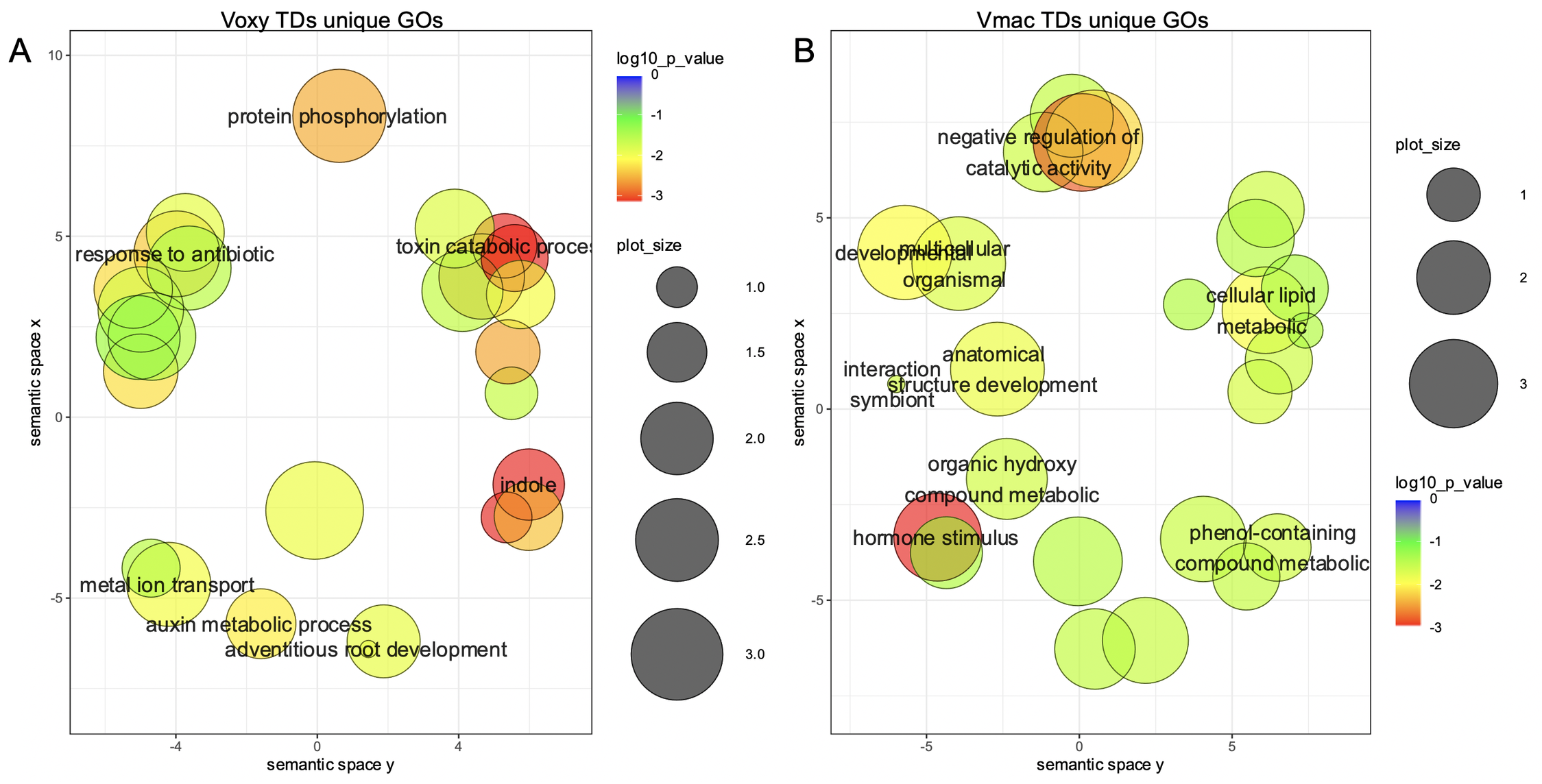

Supplement: S8 Fig — A) V. oxycoccos (Voxy) TDs unique GOs are plotted in semantic space. B) Vmac TDs unique GOs are plotted in semantic space. Significance is colored with red being the most significant and blue the least significant. The size of the circle represents the number of elements. (TIF) [file pone.0264966.s008.tif]

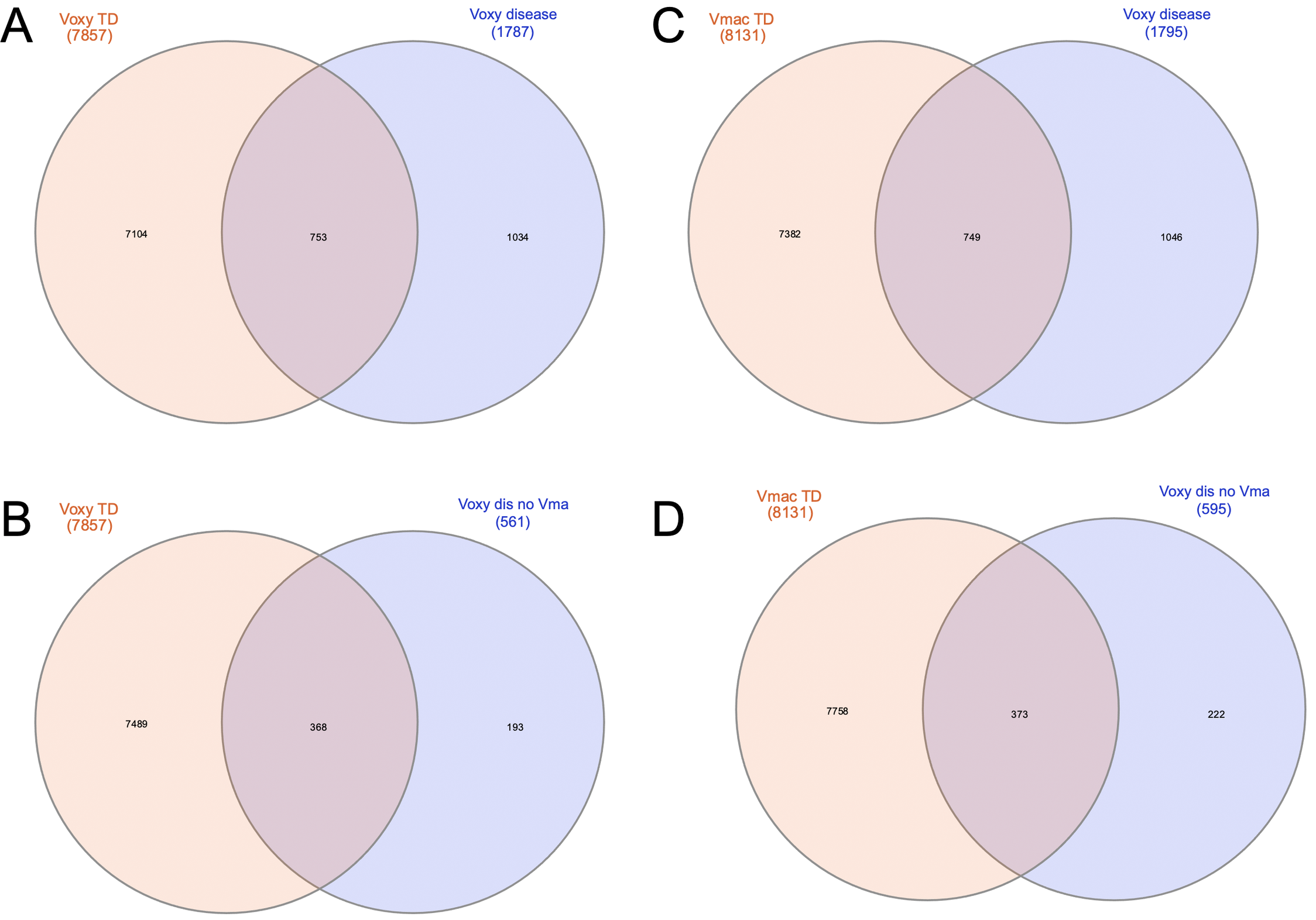

Supplement: S9 Fig — A) Voxy TD overlaps with predicted disease resistance genes, and B) disease resistance genes specific to Voxy (no syntenic ortholog in Vmac). C) Vmac TD overlaps with predicted disease resistance genes, and D) disease resistance genes specific to Vmac (no syntenic ortholog in Voxy). (TIF) [file pone.0264966.s009.tif]

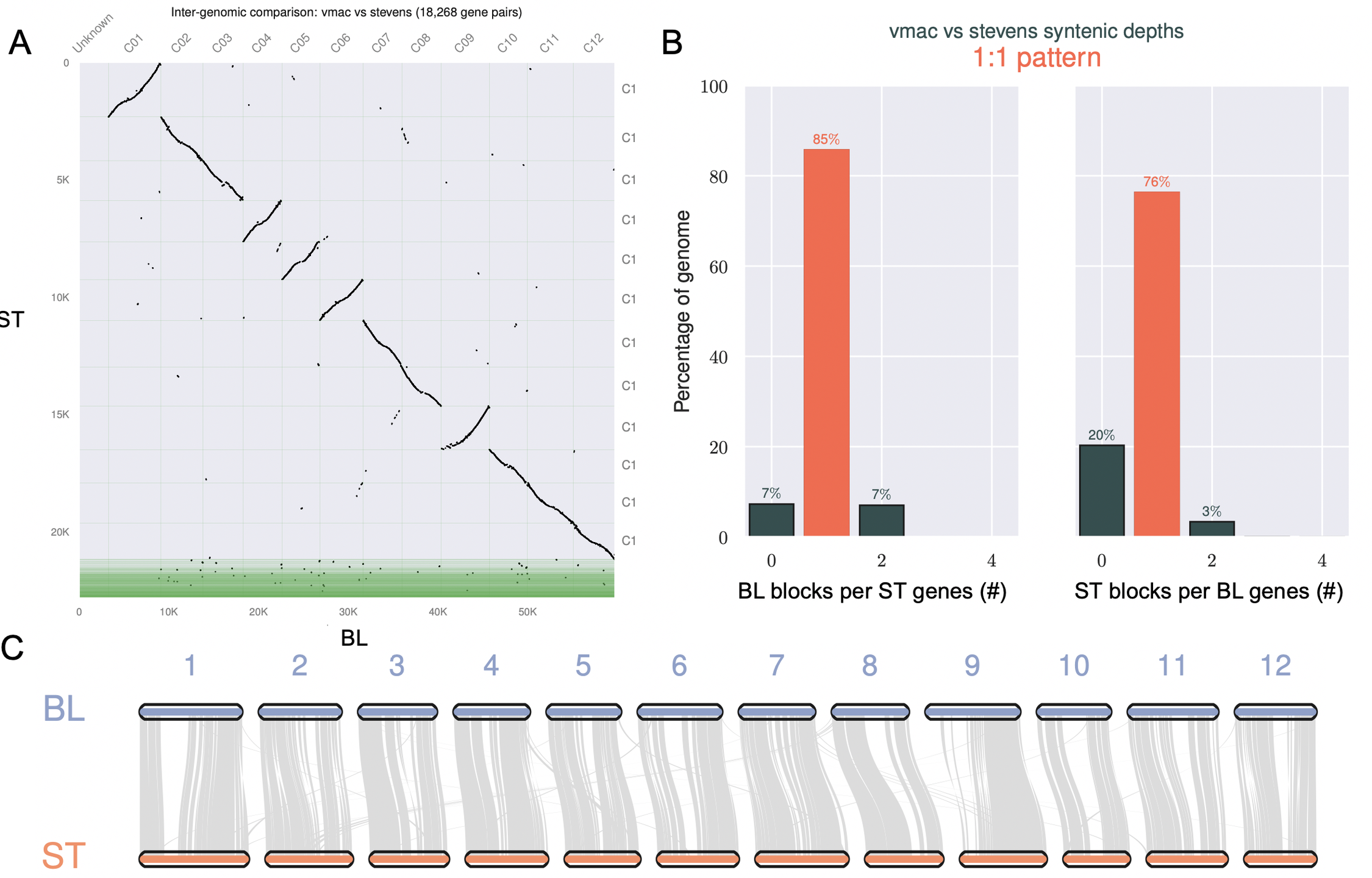

Supplement: S10 Fig — A) Dotplot between BL and ST based on protein-protein comparisons reveals differences in chromosome size between the two access but high collinearity. Green area for ST are the contigs not included in the chromosomes. B) Syntenic ortholog patterns between BL and ST reveals that the ST genome is more fragmented than the BL genome due to more (20% vs 7%) genes with zero (0) syntenic blocks. C) Chromosome alignment between BL and ST with grey lines representing syntenic blocks. The missing regions between the two assemblies are centromere and repeat regions missing in ST. (TIF) [file pone.0264966.s010.tif]
